# ACE2/ADAM17/TMPRSS2 Interplay May Be the Main Risk Factor for COVID-19

**DOI:** 10.3389/fimmu.2020.576745

**Published:** 2020-10-07

**Authors:** Donato Zipeto, Julys da Fonseca Palmeira, Gustavo A. Argañaraz, Enrique R. Argañaraz

**Affiliations:** ^1^Department of Neuroscience, Biomedicine and Movement Sciences, University of Verona, Verona, Italy; ^2^Laboratory of Molecular Neurovirology, Faculty of Health Science, University of Brasília, Brasilia, Brazil

**Keywords:** ADAM17, ACE2, TMPRSS2, SARS-CoV-2, COVID-19 pathophysiology

## Abstract

The Coronavirus Disease 2019 (COVID-19) has already caused hundreds of thousands of deaths worldwide in a few months. Cardiovascular disease, hypertension, diabetes and chronic lung disease have been identified as the main COVID-19 comorbidities. Moreover, despite similar infection rates between men and women, the most severe course of the disease is higher in elderly and co-morbid male patients. Therefore, the occurrence of specific comorbidities associated with renin–angiotensin system (RAS) imbalance mediated by the interaction between angiotensin-converting enzyme 2 (ACE2) and desintegrin and metalloproteinase domain 17 (ADAM17), along with specific genetic factors mainly associated with type II transmembrane serine protease (TMPRSS2) expression, could be decisive for the clinical outcome of COVID-19. Indeed, the exacerbated ADAM17—mediated ACE2, TNF-α, and IL-6R secretion emerges as a possible underlying mechanism for the acute inflammatory immune response and the activation of the coagulation cascade. Therefore, in this review, we focus on the main pathophysiological aspects of ACE2, ADAM17, and TMPRSS2 host proteins in COVID-19. Additionally, we discuss a possible mechanism to explain the deleterious effect of ADAM17 and TMPRSS2 over-activation in the COVID-19 outcome.

## Introduction

The Severe Acute Respiratory Syndrome Coronavirus Virus 2 (SARS-CoV-2), which first appeared in Wuhan, Hubei province, China (1, 2), spread rapidly around the world, leading to a pandemic scenario and causing thousands of deaths (2). The higher mortality and morbidity rates in coronavirus disease 19 (COVID-19) are associated with comorbidities, such as cardiovascular disease, pulmonary hypertension, diabetes, liver, and kidney diseases (3). An important bias, found in several studies conducted in different countries, is that the most severe course of the disease occurs mainly in elderly male individuals, despite similar infection rates between men and women (4–6). Curiously this disease sex bias has also been observed in SARS-CoV and MERS outbreaks (7, 8), highlighting similarities in the pathogenic mechanisms between these viruses (9). These observations provide significant indications of a possible genetic predisposition to the viral infection and the severity of the disease.

In this review, we introduce key pathophysiological concepts related to the interplay between the angiotensin-converting enzyme 2 (ACE2), desintegrin and metalloproteinase domain 17 (ADAM17) and the type II transmembrane serine protease (TMPRSS2) in the major clinical disorders reported in COVID-19 patients.

## SARS-CoV-2 Infection

The coronavirus surface spike (S) protein mediates the entry of SARS-CoV-2 into the target cells by binding to ACE2 and the subsequent fusion of the viral envelope with the host cell membrane (10, 11). After binding of SARS-S to ACE2, the S-protein undergoes a proteolytic cleavage between the S1 and S2 subunits by the TMPRSS2 serine protease (12–14). Alternatively, another less important pathway involves cathepsin L, a pH-dependent endo-lysosomal host cell protease, after uptake of virions into target cell endosomes (15). Entry through the non-endosomal pathway is more efficient than the endosomal pathway, showing the greater relevance of the protease-dependent cleavage (16). However, data from an *in vitro* experiment blocking the two proteases suggest the involvement of an additional protease (17). The furin protease emerges as a likely candidate, since the SARS-CoV-2 S-protein contains four redundant furin cleavage sites that are absent in the SARS-CoV (18). In addition, the new coronavirus has an S1/S2 cleavage site (RRARSVAS) similar to a host furin-cleavable peptide in epithelial sodium channel α-subunit (ENaC-α) (19). Moreover, sequence-based prediction studies suggest a more efficient cleavage of the SARS-CoV-2 than the SARS-CoV S-protein by furin (18, 20). These differential characteristics may explain the higher SARS-CoV-2 infectivity. However, Xia and colleagues have recently demonstrated, in an *in vitro* assay using 293T cells, that furin cleavage sites might not be very relevant for SARS-CoV-2 infections in human airway (21). In addition, a neutrophil elastase (NE) cleavage site near the S1–S2 protein was recently identified in the A2a SARS-CoV-2 subtype (D614G mutation), suggesting an important role of neutrophil elastase in the infection (22). Therefore, the possible participation of other proteases in the viral entry requires further investigation.

## ACE2/ADAM17 in COVID-19’s Comorbidities

ACE2 is a type I transmembrane, endothelium-bound carboxymonopeptidase protein, and ubiquitously expressed in endothelial cells of several organs, with the highest levels in the cardiovascular system, intestine, kidneys, brain, testicles and lungs (23). The ACE2 gene is located on the X-chromosome and is highly polymorphic (24). Due to its genomic location, ACE2 expression could be affected by parental imprinting and X-inactivation in females, resulting in different ACE2 expression levels and renin activation between females and males (25, 26). Furthermore, the possible involvement of ACE2-related genetic factors in the pathogenesis of COVID-19 has been supported by the identification of polymorphic markers in the ACE2 locus and present in patients with specific comorbidities related to the severity of COVID-19 (5, 27, 28).

The ACE2 molecule, besides being a receptor of SARS-CoV and SARS-CoV-2 (29–32), reduces the activity of the renin–angiotensin system (RAS) by converting angiotensin (Ang) I and Ang II into Ang 1-9 and Ang 1-7 respectively (33–35). Thus, the ACE2 protein has been shown to play an important role in protecting against some disorders such as cardiovascular complications, chronic obstructive pulmonary disease (COPD) and diabetes, among other COVID-19 comorbidities (36). The ACE2/Ang 1-7 axis counterbalances the ACE/Ang II-I axis by decreasing Ang II levels, the activation of angiotensin type 1 receptors (AT1Rs) and, thus, leads to decreased pathophysiological effects on tissues, such as inflammation and fibrosis (37). Furthermore, it is important to note that the difference in ACE2 expression levels also depend on factors such as age and lifestyle. There are evidences that ACE2 activity differs between males and females (38), with males having higher levels in the lungs (39). Recent studies have shown an increase in plasma levels of the soluble form of ACE2 (sACE2) with age, being higher in men than in women (40). This increase was interpreted, in some cases, as a consequence of an increased activity of ADAM17-sheddase (will be discussed below). Moreover, using public gene expression datasets, a differential expression was found not only for ACE2 but also for the TMPRSS2 gene in the nasal and bronchial airways in relation to age (41). Interestingly, it was found a higher TMPRSS2 expression on nasal epithelial cells from Black individuals than Asian, Latino, and White individuals (42). These finding could explain the 2–3 times higher incidence of COVID-19 among Black individuals (43).

Children have shown significantly lower expression of both SARS-CoV-2 receptors in the upper and lower airways (41). Regarding ACE2 activity, some studies have shown that ACE2 activity is lower in older women than in young ones, while the same does not occur in males (38, 44). These findings indicated that the regulation of ACE2 expression may be the result of a process dependent on both age and gender, and may be related to the pathological progression and poor prognosis of COVID-19 (45).

As far as lifestyle is concerned, two habits seem to have a significant effect on ACE2: cigarette smoking and diet. The former has been shown to decrease ACE2 blood levels (45), leading to an imbalance in RAS homeostasis (46). On the other hand, Smith and colleagues showed that cigarette smoking causes a dose-dependent upregulation of ACE2 both in rodent and human lungs (47). These findings were recently confirmed by Sharif-Askariboth and colleagues, who observed a significant ACE2 upregulation in the nasal and bronchial airways of smokers compared to non-smokers (41). Two systematic reviews by Zhao et al. and Vardavas & Nikitara, indicate that smoking is indeed a risk factor in COVID-19 (48, 49). Apparently, nicotine promotes ACE2 overexpression through the alpha7-nicotinic receptor (50). However, there are many other substances in cigarette smoke that promote respiratory and cardiovascular diseases and need to be investigated. On the other hand, considering that the expression of ACE2 may promote protection against pulmonary and cardiovascular damage, it is plausible to think that both functions of ACE2 may play a role in COVID-19, however, at different times of the infection process and with different impact. Diet has been associated with the modulation of ACE2 expression and its activity. Indeed, salt rich diets tend to increase ACE2 activity (51) and the rate of ACE/ACE2 in the glomeruli (52), while glucose rich diets may decrease ACE2 leading to an imbalance between ACE/ACE2 in the heart (53).

Concerning to ADAM17 proteinase, it is expressed by several tissues, such as muscle, lungs, placenta, ovaries, testicles, pancreas, kidney, small intestine, thymus, and heart (54). As a membrane “sheddase” protease, ADAM17 removes the membrane protein ectodomains (sheddase activity). The mechanisms by which ADAM17 is regulated have not yet been fully elucidated, but would depend on the stimulus and the cell type. Specifically, in the ADAM17-mediated shedding of the soluble active TNF-α form and of the epidermal growth factor receptor (EGFR), the participation of iRhom2, a proteolytically inactive member of the rhomboid family, proved to be important for maturation and substrate specificity of ADAM17 (55). On the other hand, ADAM17 activity could be induced by apoptosis, Ca^2+^ ionophores, fibroblast growth factor 7 (FGF7), protein kinase C (PKC) activators and purine 2 (P2) receptor agonists (56–59). In addition, ADAM17 can also be activated in response to pathogen infection through Toll-like receptors (60, 61) and may involve the translocation of phosphatidylserine (PtdSer) to the outer cell membrane leaflet, a crucial step for ADAM17 to exert its “sheddase” activity (62).

### ACE2/ADAM17 in Cardiovascular Disease

In the human and animal model of cardiovascular disease and hypertension, an increase in the ACE/AC2 ratio has been reported, as a consequence of an increased downregulation of ACE2 expression (63–65). Moreover, the activity of the soluble form of ACE2 (sACE2) is increased in patients with heart failure (HF) and correlates with the severity of the disease (65–67). The relevance of RAS unbalance in cardiovascular diseases has been highlighted by a recent study of Sama and colleagues (67). In this study, men with heart failure had higher ACE2 plasma levels than women in the same condition. Thus, increased ACE2 plasma levels have been interpreted as a molecular marker of a poor prognosis. These authors did not find an association between the use of ACE inhibitors and angiotensin receptor blockers (ARBs) with higher plasma ACE2 concentration plasma levels (67). These findings suggest that the use of ACE2 inhibitors and ARBs would not increase the vulnerability to a poor clinical outcome in patients with COVID-19. Finally, and based on the impact of the use of ACE inhibitors, AT1Rs blockers and mineralocorticoid receptor antagonists, the higher plasma levels of ACE2 have been interpreted as a consequence of the higher activity of ADAM17 (68). In support of this interpretation, elevated levels of TNF-α have been found in patients with heart disease, such as myocarditis, and were negatively correlated with left ventricular systolic function (69).The increased expression of ADAM17 was reported in peripheral blood mononuclear cells (PBMC) in patients with congestive heart failure, as well as in malignant recurrent ventricular arrhythmia after acute myocardial infarction (70, 71). Moreover, the increased expression of ADAM17, as well as of TNF-α was associated with rupture of atherosclerotic coronary plaques in patients with myocardial infarction (72). Finally, ADAM17 knockdown inhibited hypertension-induced cardiac hypertrophy and fibrosis (73).

In summary, several lines of evidence support that low expression levels of the ACE2 membrane form (mACE2) and higher ADAM17 activity would be associated with cardiovascular disease. In the context of SARS-CoV-2 infection this condition would be even more exacerbated as a consequence of a molecular mechanism triggered by SARS-CoV-2 infection (74), thus explaining the higher incidence of COVID-19 in these kind of patients (68).

### ACE2/ADAM17 in Pulmonary Disease

ACE2 has protective effects in the lungs (63). ACE2 knockout results in a pathology similar to the acute respiratory distress syndrome (ARDS) in mice (75), and ACE2 polymorphisms have been correlated with the ARDS severity (29). Moreover, ACE2 has been shown to protect mice with acute lung disease by SARS-CoV infection (76). In this study genetic evidence confirmed that ACE2 is a crucial SARS-CoV receptor *in vivo*, and that the Spike protein of the SARS-CoV reduces ACE2 expression. These results provided a molecular explanation of why SARS-CoV infections cause severe and often lethal lung failure and suggest a rational for the use of an ACE2 replacement as therapy.

Among the lung diseases associated with the most severe clinical picture of COVID-19 are chronic obstructive pulmonary disease (COPD) and cystic fibrosis (CF) (77). COPD is characterized by emphysema, chronic bronchitis and irreversible airflow obstruction due to airway inflammation. Analysis of transcriptomic data of biopsies from COPD patients have shown a significantly upregulation of ACE2 gene expression when compared with healthy subjects. These results are correlated with the upregulated ACE2 gene expression in the lung tissues of smokers compared to healthy individuals (41). In addition, the modulation of the ADAM17 and EGF receptor (EGFR) axis is another important factor involved in COPD and CF, to maintain the balance between anti and pro inflammatory processes and excessive tissue regeneration and remodeling. Indeed, the EGFR/ADAM17 signaling pathway can exercise anti- and pro- inflammatory functions, depending on stimuli, substrate and cell type (78). In this sense, pathogens as virus and bacteria can activate the ADAM17/EGFR signaling pathway through Toll-like receivers (TLR) (60, 61). The ADAM17/EGFR - mediated pro-inflammatory response is mediated by the neutrophil chemotactic factor CXCL8 (79), and the anti-inflammatory response through the shedding of TNF receptor type 2 (TNFR2). Furthermore, Stolarczyk and colleagues (80) showed a higher ADAM17 mediated, pro-inflammatory molecules secretion, such as CXCL8, the IL6 cytokine receptor (IL6R) and growth factor amphiregulin (AREG) in airway epithelial cells from COPD patients by cigarette smoke (CS). The subsequently activation of interleukin receptor gp130 and EGFR leads to lung inflammation, repair, sub-epithelial fibrosis and collagen deposition. Therefore, the reduction of ADAM17 and EGFR activity may contribute to anti-inflammation and tissue remodeling in COPD (80). Finally, these data may explain why patients with COPD infected by SARS-CoV-2 are more susceptible to progress towards severe symptoms of COVID-19 (81).

### ACE2/ADAM17 in Diabetes and Renal Diseases

Both ACE2 and ADAM17 have functions associated with glycemic homeostasis. ACE2 modulates insulin secretion from pancreatic β-cells and the growth of pancreatic islet cells (63). In addition, studies have demonstrated the protective role of ACE2 against pancreatic dysfunction in diabetes and that ACE2 is related to diabetic nephropathy (DN) (63). On the other hand, the increased activity of ADAM17 has been correlated with increased insulin resistance and hyperglycemia (82, 83). Furthermore, the upregulation of ADAM17 activity exacerbated tissue damage in DN (84) and increased insulin receptor resistance in patients with type 2 diabetes (85, 86). In addition, insulin treatment has been shown to attenuate renal secretion of ADAM17 and ACE2 in diabetic mice, suggesting that tubular renal secretion of ACE2 could be mediated by ADAM17 in diabetes-induced type 1 nephropathy.

ACE2 has a protective effect through the regulation of RAS (87), and plays a crucial role in the pathogenesis of renal diseases, decreasing levels in acute kidney injury and chronic renal disease induced by hypertension and diabetes (88). In this context, the worse clinical outcome observed in COVID-19 patients with diabetes may be due to the exacerbated decline in ACE2 induced by the up-regulation of ADAM17 as consequence of SARS-CoV-2 infection.

Renal dysfunction is one of the most serious complications in patients with COVID-19 (81) with a high risk of mortality. ACE2, TMPRSS2, and cathepsin L are highly expressed and active in the kidney (89, 90). In retrospective, postmortem tissue studies from COVID-19 patients detected SARS-CoV-2 viral load in the kidney, with preference in glomerular cells and tubules. Moreover, SARS-CoV-2 infection more frequently induced acute renal failure in elderly patients with comorbidities such as hypertension and heart failure (90, 91).

## ACE2/ADAM17/TMPRSS2 Interplay in COVID-19

ACE2 plays an important role in SARS-CoV-2 infection not only in viral entry (30–32), but also in providing protection against acute cardiovascular and pulmonary injury and protection against DIC caused by COVID-19 (37, 92, 93). After interaction with the viral S-protein, ACE2 is internalized along with viral particles into endosomes, reducing surface tissue expression of ACE2. Interestingly, after SARS-CoV infection of myocardial and lung tissues, the ACE2 mRNA expression is also inhibited (76, 94, 95).

On the other hand, the ADAM17 activity is up-regulated by the internalization of SARS-CoV–ACE2 in the ACE2 cytoplasmic dependent manner (74). The ADAM17 up-regulation leads to the ACE2 ectodomain proteolytic cleavage (65, 96, 97). Curiously, the ACE2 tail as well as ADAM17 expression proved to be necessary for SARS-CoV infection. However, the underlying mechanism by which the interaction of the S/ACE2 proteins mediates ADAM17 activation is not yet clear, nor how ADAM17 facilitates viral entry. Probably, the protease activity of ADAM17 might be important for the fusion of viral particles with the cytoplasmic membrane. Finally, the increased activity of the ADAM17 sheddase may also lead to the cleavage of TNF-α and IL6R and other pro-inflammatory molecules, reinforcing the inflammatory process during SARS-CoV-2 infection (37, 98). ACE2 can also be cleaved by the TMPRSS2 protease (97). TMPRSS2-mediated ACE2 cytoplasmic tail cleavage may lead to an increased viral uptake and processing through a cathepsin L-dependent pathway (97).

In this context, it is important to highlight that the internalization of ACE2 can trigger positive feedback harmful to tissues. The ACE2 downmodulation would lead to an increase in Ang II level and the activation of the A1T receptor, which in turn would activate ADAM17 more. Therefore, the exacerbated reduction of ACE2 is, indirectly, responsible for its own cleavage by ADAM17 and, consequently, for the depletion of ACE2 in the tissue and release of the sACE2 form (68). The sACE2 and mACE2 are composed by extracellular and membrane bound domains of ACE2, respectively. Additionally, the ACE2 expression could also be transcriptionally downmodulated as a consequence of AT1Rs activation (99). Indeed, tissue downregulation of ACE2 mediated by the SARS-CoV-2 infection reduces the protective effects of ACE2 in various organs such as kidneys, heart, lungs and gut through Ang 1-7 which counteracts the Ang II effects (34, 36, 100). The beneficial effects of recombinant ACE2 administration on human pulmonary arterial hypertension (101) and in lung lesions caused by viral infections (102, 103), support the relevance of sACE2 in blocking viral particles in preventing the interaction with transmembrane ACE2. The marked reduction of ACE2 expression on the cell surface causes an imbalance in the RAS system, leading to an increase in Ang II plasma levels, which in turn promote thrombosis, through a thrombin-dependent pathway (104). Ang II does not just act as a vasoconstrictor but also as a pro-inflammatory molecule through AT1R (105). Indeed, the Ang II-AT1R axis activates ADAM17 which in turn releases the mature form of EGFR, the soluble form of IL-6Ra (sIL-6Ra) and TNF-α (105, 106). On the other hand, endothelial cells infection and activation of macrophages in the pulmonary alveoli are the main source of pro-inflammatory cytokines such as IL-1b, IL-6, IFN-γ, IL-8, and TNF-α triggering the acute inflammatory response, known as “cytokine storm” (107, 108). These inflammatory infiltrates in the alveolar lumen release toxic molecules leading to diffuse alveolar damage, pulmonary edema and fibrin deposition in the alveolar space (109). Moreover, endothelial injury has also been associated with elevated plasma level of von Willebrand factor (vWF), neutrophils and macrophages infiltrate (110). Afterwards, activated neutrophils secrete neuthrophil extracellular traps (NETs), increasing (110) endothelium damage and activation of both the extrinsic and intrinsic pathway (111). Finally, the prothrombotic state could be facilitated by hypoxia-inducible factor (HIF) after lung injury (112).

## ACE2/ADAM17/TMPRSS2 Underlying Risk Factors for COVID-19

The involvement of ACE2 in the pathophysiology of COVID-19 has become a “conundrum” that needs to be unraveled. While ACE2 is the viral receptor for SARS-CoV-2, it also provides protection against the harmful effects of RAS-mediated activation by viral infection (37). Moreover, as mentioned above, strong evidences indicate that the expression of ACE2 would be subject to mechanisms of hormonal, genetic and age-related regulations (5, 40, 113, 114).

Several data support the involvement of an increased activity of ADAM17 in both COVID-19`s comorbidities and SARS-CoV-2 infection: i) increased plasma levels of ACE2 soluble form (sACE2) with age in men (40, 41); ii) higher expression levels of sACE2 in men, with heart failure, than woman as a consequence of increased ADAM17 activity (67); and iii) higher ACE2 level expression and up-regulated activity of ADAM17 in patients with chronic pulmonary inflammation (54), COPD (41, 80), diabetes (82, 83) and renal diseases (86). These findings support the epidemiological data showing a higher incidence and severity of COVID-19 in elderly men with comorbidities (115) than in healthy young men or women (4, 6). In fact, while the rate of fatal cases in patients without comorbidities is approximately 0.9%, it is much higher for patients with COVID19’s comorbidities: specifically, cardiovascular disease (10.5%), diabetes (7.3%), and hypertension (6.3%) (116).

In this context, a key factor to understand this apparent paradox in the pathophysiology of COVID-19, related to the conflicting evidences ACE2’s role, could be found in the delicate interplay balance between the cleavage of ACE2 by both ADAM17 and TMPRSS2 proteases in a stepwise fashion.

**Figure 1:**
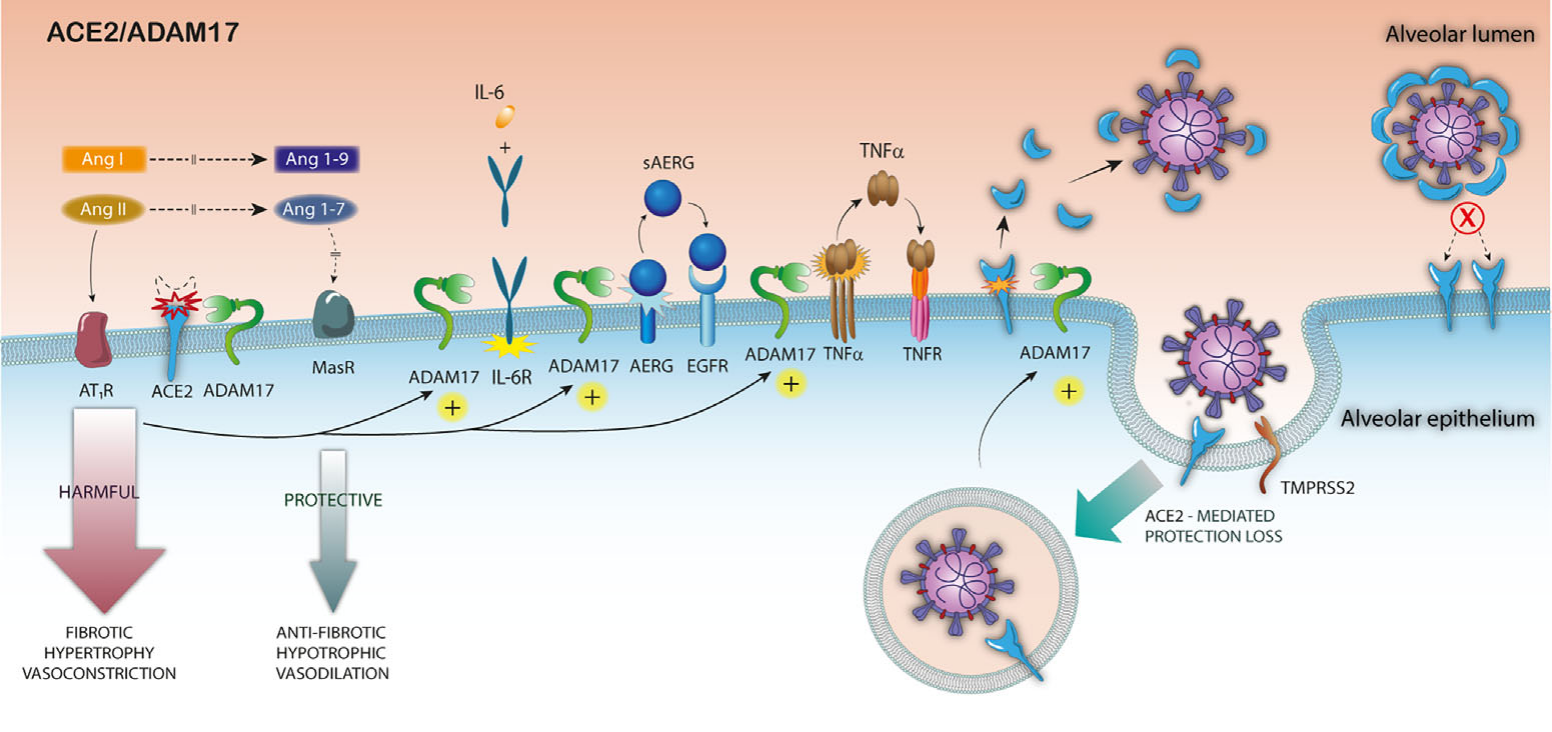
Pathophysiological consequences of ADAM17 over-activation in SARS-CoV-2 infection. After the binding of SARS-S to ACE2, the S-protein undergoes a proteolytic cleavage by TMPRSS2, and the virus enters the host cell. The attachment of the S protein to ACE2 triggers ADAM17 activation, increasing mACE2 downmodulation, reducing surface ACE2 expression. The increased ADAM17-mediated ACE2 shedding exacerbates the imbalance of RAS, in a looping feedback manner and increases inflammation by TNF-α the IL6 cytokine receptor (IL6R) and growth factor amphiregulin (AREG) cleavage. Finally, the sACE2 release by ADAM17 cleavage might block the viral particles entry.

At the initial infection stage, the high mACE2 expression levels in nasal and bronchial airways and lung in patients with some comorbidities or harmful lifestyles for COVID-19 may be a critical risk factor for higher infection levels (41, 69, 117). Posteriorly, and as a consequence of the high viral infection level, the expression of ACE2 and its mRNA would be greatly diminished in these patients. Moreover, the SARS-S/ACE2 interaction would trigger the ACE2 cytoplasmic tail alteration, which would induce ADAM17 activation and viral entry (**Figure 1**). On the other hand, the increased expression of IL-1β, as a consequence of the inflammatory process induced by SARS-CoV-2 infection, may also contribute to the over-activation of ADAM17 in the early stage of the infection (118).

At a later stage, the SARS-S/ACE2 mediated up-regulation of ADAM17 in pulmonary and cardiac tissues may exacerbate the imbalance in RAS, already present in patients with risk diseases in a looping harmful feedback manner, thus becoming the main responsible for the worst clinical outcome. The exacerbated up-regulation of ADAM17-mediated ACE2 cleavage would lead to two additional harmful effects: i) prevent the premature infected cells death by superinfection-mediated apoptosis, before viral particles release; ii) preclude the virus locking mediated by the interaction with surface ACE2 during viral budding. It is important to emphasize that virus-mediated receptor down-modulation strategies to optimize viral infectivity are used by other viruses, such as HIV-1 (119, 120), influenza, measles and pulmonary SARS-CoV (76) (**Figure 1**).

**Figure 2:**
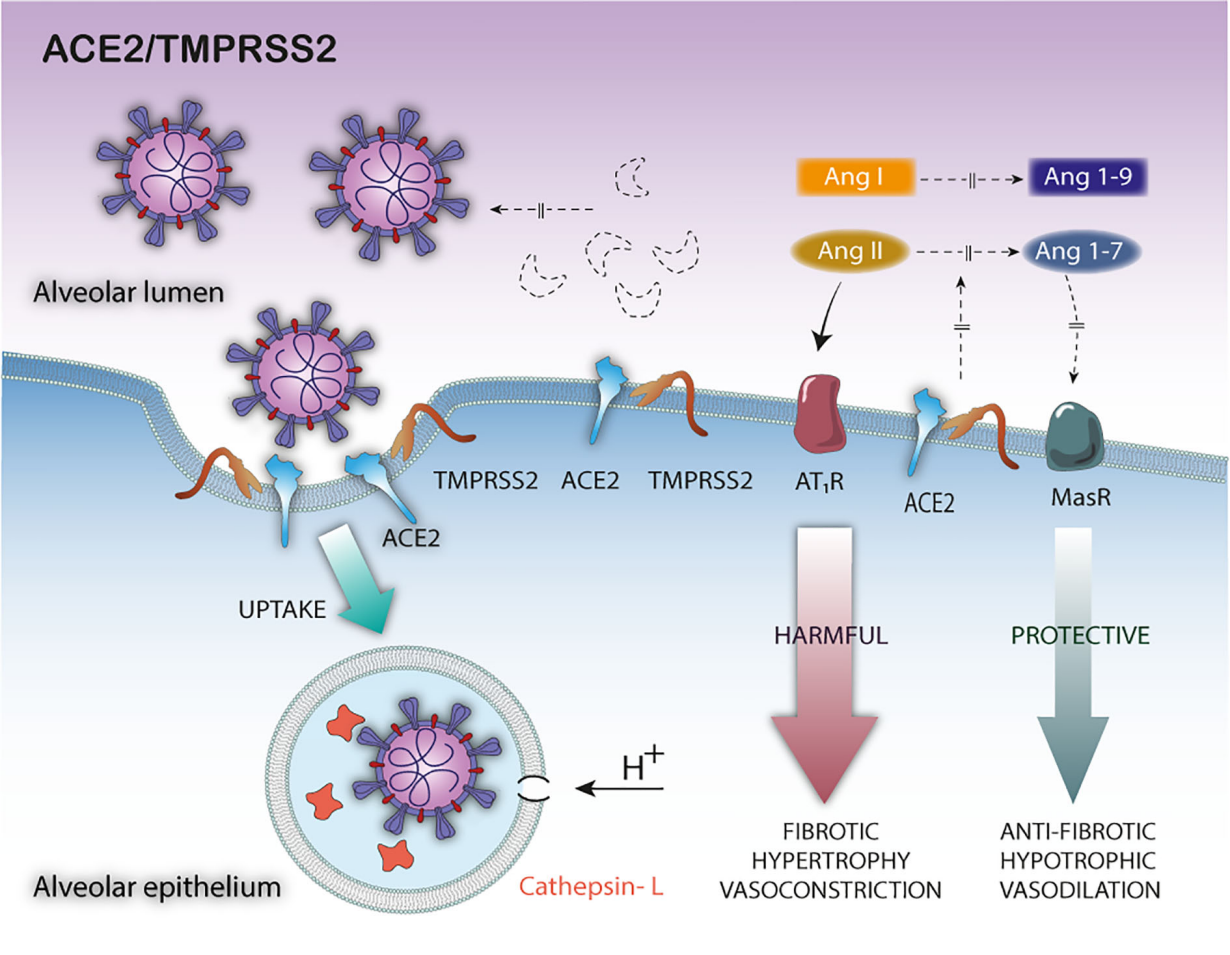
Pathophysiological consequences of TMPRSS2—mediated ACE2 cleavage in SARS-CoV-2 infection. The TMPRSS2-mediated ACE2 cleavage promotes viral uptake through the cathepsin L-dependent pathway, inactivates the ACE2 membrane-bound form leaving the tissue unprotected against detrimental effect of RAS activation and does not release the soluble blocking form of ACE2.

Another important mechanism to take into account, in this complex pathophysiological equation, is the ACE2 cytoplasmic tail cleavage mediated by TMPRSS2 (97). The cleavage of the ACE2 tail by TMPRSS2 increases viral uptake in target cells and, therefore, TMPRSS2 could promote SARS-CoV-2 entry by two mechanisms: i) by SARS-S cleavage, which activates the S protein for membrane fusion, and ii) by ACE2 cleavage, which might promote viral uptake through the cathepsin L-dependent pathway, which would then infect the cell by fusing with the endosomal membrane (97) (**Figure 2**).

Although TMPRSS2 and ADAM17 share the same specificity for ACE2, important differences can be observed in the cleavage of ACE2 (97). First, the fractions generated by the cleavage of these proteases have different molecular sizes due to different cleavage sites. Second, the ACE2 ectodomain released by the cleavage by ADAM17 is shed into the extracellular medium, as sACE2 biologic active form. However, the TMPRRS2-mediated ACE2 cleavage would not release the ACE2 ectodomain; instead, a C-terminal intracellular cleavage fragment was observed, at least as demonstrated by *in vitro* experiments (97) (**Figure 2**). Therefore, the differences in the cleavage sites and its biological consequences may be critical. Indeed, only the sACE2 form would exert a protective effect, blocking circulating viral particles (76) (**Figure 1**). In short, although the cytoplasmic tail cleavage of ACE2 by TMPRSS2 and ADAM17 act synergistically unprotecting the pulmonary and cardiac tissue against the harmful effects of RAS activation, only the TMPRSS2-mediated cleavage would not release the ACE2 soluble active forms. Thus, the TMPRSS2 - mediated ACE2 cleavage would be more harmful to the host. However, it is still unknown whether ACE2 cleavage by both proteases is mutually exclusive or not, and the relevance of the interplay of these mechanisms on the pathophysiology of COVID-19. In this scenario, it can be expected that increased expression of TMPRSS2 induced by androgen hormones or the occurrence of specific genetic variants (5, 42, 121), or both factors together, may lead to exacerbate, even more, the ACE2 cleavage—mediated harmful effects, by decreasing the membrane-bound or soluble biologically active ACE2 forms.

This hypothesis may explain the higher incidence in men than in women or in certain races (5, 42, 121). More recently, a specific SARS-CoV-2 A2a subtype—mediated TMPRSS2 and MX1 genes up-regulation has been described in European and North American populations (22). These findings along with the higher TMPRSS2 expression on nasal epithelial cells from Black individuals than others races, may explain the higher rates of infection, and worse COVID-19 clinical outcomes observed in these populations, by the increasing TMPRSS2-mediated SARS-CoV-2 and ACE2 cleavage.

In this context, it is plausible to assume that the medical history of COVID-19 patients, in terms of comorbidities, sex, age, race and specific life style associated with high mACE2 expression levels in the upper airways and lung, may be a risk factor for higher infection levels at the first stage of the infection. Subsequently, an exacerbated decrease in the membrane-bound active ACE2 form, both for ADAM17 and TMPRSS2 over-activation, and for the infection itself, may exacerbate the RAS imbalance (ACE/ACE2) in patients with specific comorbidities, such as cardiovascular disease, hypertension, diabetes and chronic lung disease. Finally, the over-activation of TMPRSS2 in elderly and co-morbid male patients may be decisive for the clinical outcome, leading to an acute setting rather than a moderate clinical outcome. Therefore, over-activation of ADAM17 and TMPRSS2 could be the main mechanism underlying the negative effects of RAS imbalance, acute inflammation and intravascular coagulation observed in elderly males with COVID-19 comorbidities.

## Concluding Remarks

In the context of the current pandemic, it is a priority to gain a detailed understanding of the molecular mechanisms involved in the pathophysiology of COVID-19 in order to identify risk factors and develop new effective therapeutic approaches. The evidence presented in this review highlights the deleterious effect of ACE2 downmodulation by ADAM17 and TMPRSS2 in COVID-19 pathogenesis. The delicate balance between ACE2, ADAM17, and TMPRSS2 interactions in each specific pathophysiological condition appears as a critical key factor. Therefore, the occurrence of specific comorbidities associated with RAS imbalance mediated by ACE2/ADAM17 along with specific genetic factors mainly associated with TMPRSS2 expression could be decisive for the clinical outcome of COVID-19.

## Author Contributions

All authors listed have made a substantial, direct, and intellectual contribution to the work and approved it for publication.

## Conflict of Interest

The authors declare that the research was conducted in the absence of any commercial or financial relationships that could be construed as a potential conflict of interest.
